# Fertilization Shapes Bacterial Community Structure by Alteration of Soil pH

**DOI:** 10.3389/fmicb.2017.01325

**Published:** 2017-07-18

**Authors:** Yuting Zhang, Hong Shen, Xinhua He, Ben W. Thomas, Newton Z. Lupwayi, Xiying Hao, Matthew C. Thomas, Xiaojun Shi

**Affiliations:** ^1^College of Resources and Environment, Southwest University Chongqing, China; ^2^Agriculture and Agri-Food Canada, Lethbridge Research and Development Centre Lethbridge, AB, Canada; ^3^Centre of Excellence for Soil Biology, College of Resources and Environment, Southwest University Chongqing, China; ^4^School of Biological Sciences, University of Western Australia Crawley, WA, Australia; ^5^Calgary Laboratory, Canadian Food Inspection Agency Calgary, AB, Canada; ^6^Academy of Agricultural Sciences, Southwest University Chongqing, China

**Keywords:** 16S rRNA gene, 454 pyrosequencing, acidification, eutric regosol, nutrient availability

## Abstract

Application of chemical fertilizer or manure can affect soil microorganisms directly by supplying nutrients and indirectly by altering soil pH. However, it remains uncertain which effect mostly shapes microbial community structure. We determined soil bacterial diversity and community structure by 454 pyrosequencing the V1-V3 regions of 16S rRNA genes after 7-years (2007–2014) of applying chemical nitrogen, phosphorus and potassium (NPK) fertilizers, composted manure or their combination to acidic (pH 5.8), near-neutral (pH 6.8) or alkaline (pH 8.4) Eutric Regosol soil in a maize-vegetable rotation in southwest China. In alkaline soil, nutrient sources did not affect bacterial Operational Taxonomic Unit (OTU) richness or Shannon diversity index, despite higher available N, P, K, and soil organic carbon in fertilized than in unfertilized soil. In contrast, bacterial OTU richness and Shannon diversity index were significantly lower in acidic and near-neutral soils under NPK than under manure or their combination, which corresponded with changes in soil pH. Permutational multivariate analysis of variance showed that bacterial community structure was significantly affected across these three soils, but the PCoA ordination patterns indicated the effect was less distinct among nutrient sources in alkaline than in acidic and near-neural soils. Distance-based redundancy analysis showed that bacterial community structures were significantly altered by soil pH in acidic and near-neutral soils, but not by any soil chemical properties in alkaline soil. The relative abundance (%) of most bacterial phyla was higher in near-neutral than in acidic or alkaline soils. The most dominant phyla were Proteobacteria (24.6%), Actinobacteria (19.7%), Chloroflexi (15.3%) and Acidobacteria (12.6%); the medium dominant phyla were Bacterioidetes (5.3%), Planctomycetes (4.8%), Gemmatimonadetes (4.5%), Firmicutes (3.4%), Cyanobacteria (2.1%), Nitrospirae (1.8%), and candidate division TM7 (1.0%); the least abundant phyla were Verrucomicrobia (0.7%), Armatimonadetes (0.6%), candidate division WS3 (0.4%) and Fibrobacteres (0.3%). In addition, Cyanobacteria and candidate division TM7 were more abundant in acidic soil, whereas Gemmatimonadetes, Nitrospirae and candidate division WS3 were more abundant in alkaline soil. We conclude that after 7-years of fertilization, soil bacterial diversity and community structure were shaped more by changes in soil pH rather than the direct effect of nutrient addition.

## Introduction

In agroecosystems, it is well established that soil microbial communities respond to N, P and/or K fertilizer (Allison and Martiny, 2008; Beauregard et al., 2010), manure, compost (Hartmann et al., 2015; Francioli et al., 2016) and their combinations (Sun et al., 2015; Chen et al., 2016). Mineral and organic fertilizers typically increase soil microbial biomass by supplying nutrients and/or carbon (C) to the soil microbiome (Diacono and Montemurro, 2010; Ninh et al., 2015). Researchers have linked changes in soil bacterial community structure and composition to nutrient availability. For instance, high nutrient availability promotes copiotrophs while nutrient-limited soil environments favor slow-growing oligotrophs (Fierer et al., 2007, 2012; Hartmann et al., 2015; Zhalnina et al., 2015). Nutrient availability depends on the nutrient source and application rate and thus the application of different nutrient sources may markedly shift the predominant microbial taxa (Fierer et al., 2007; Hartmann et al., 2015; Sun et al., 2015).

While fertilizers affect soil bacterial biomass and community composition by increasing nutrient availability, they also exert indirect effects on the microbial community by altering soil pH. For example, under simulated single N deposition or fertilization, soil bacterial richness, and diversity were negatively affected by increased N availability, while the bacterial community composition was indirectly altered due to soil acidification (Zeng et al., 2016). Sun et al. (2015) observed that reduced soil bacterial diversity following 30 years of chemical NPK fertilization could be reversed by combined applications of NPK and manure due to the pH neutralizing effect of the manure. A global meta-analysis of 64 fertilization trials attributed reduced soil microbial biomass and activity to changes in soil pH induced by ammonical fertilizer (Geisseler and Scow, 2014). Research from the Hoosfield and Park Grass Experiment at Rothamsted, UK showed that soil pH was the main edaphic property controlling microbial activity (Pietri and Brookes, 2008; Rousk et al., 2009, 2010; Zhalnina et al., 2015), and that bacterial richness and 16S rRNA copy numbers increased linearly within a soil pH range of 4.0 to 8.3 (Rousk et al., 2010). In a diverse set of ecosystems across South and North America, soil bacterial community structure was strongly shaped by soil pH at the continental scale, while differences in site-specific characteristics were poor predictors of bacterial community structure (Fierer and Jackson, 2006; Lauber et al., 2009).

As it remains uncertain how soil bacterial communities may respond to contrasting nutrient sources across a soil pH gradient, we applied chemical NPK fertilizer, composted manure, and chemical fertilizer plus composted manure to acidic, near-neutral or alkaline Eutric Regosol soil (pH 5.8, 6.8, or 8.4, respectively) to investigate the potential direct (i.e., nutrient) and indirect (i.e., pH change) effects on the bacterial community structure. The strong pH buffering capacity of the alkaline soil provided a unique opportunity to assess the direct effect of nutrient input on soil bacterial communities, forming a sharp contrast to the interactive effect of nutrient availability and pH on soil bacterial communities in acidic and near-neutral soil. The objectives of this study were (a) to compare the effects of 7 years of chemical and organic fertilizer applications on soil pH, nutrient and C concentrations, bacterial diversity and community structure across a soil pH gradient, and (b) to relate changes in soil pH and nutrient concentrations to the soil bacterial characteristics to determine the key drivers that shape soil bacterial community structure.

## Materials and methods

### Experimental location, treatments, and soil sampling

Soil samples were collected from the National Monitoring Base for Purple Soil Fertility and Fertilizer Efficiency, which was established in 2007 at Southwest University Campus in Beibei, Chongqing, China (29°48′N 106°24′E). This region has a subtropical monsoon moist climate with a mean annual temperature of 18.2°C and rainfall of 1,150 mm. Eutric Regosol soil samples characterized by different pH levels (5.8, 6.8, or 8.4, i.e., acidic, near-neutral or alkaline, respectively; Table 1) were collected from Suining (30°50′N 105°70′E, Sichuan, China), Beibei (29°48′N 106°24′E, Chongqing, China) and Jinyun Mountain (29°52′N 106°19′E, Chongqing, China), respectively. The Eurtic Regosol soil is characterized by fast physical weathering of sedimentary rocks of the Trias-Cretaceous system (IUSS Working Group, 2015) and covers approximately 2.6 × 10^5^ km^2^ of the Sichuan Basin (30°30′N, 105°30′E) in southwest China, supplying food for nearly 130 million people. For each soil pH-type there are four fertilization treatments with three replicates. As a result, a total of 36 experimental replicates or concrete-separated pools (150 × 100 × 100 cm) were constructed to grow crops in an annual maize-vegetable rotation system. The maize (*Zea mays*) cultivars varied from 2007 to 2014, while the vegetables were tuber mustard (*Brassica juncea*, cultivars varied) from 2007 to 2010 followed by Chinese cabbage (*Brassica campestris* cv. Pekinensis) from 2011 to 2014.

**Table 1:** Baseline soil chemical properties (0–20 cm depth) in 2007 and after 7 years of chemical fertilizer and/or manure application.

	**pH (H_2_O)**	**SOC (g kg^−1^)**	**TN (g kg^−1^)**	**TP (g kg^−1^)**	**TK (g kg^−1^)**	**AN (mg kg^−1^)**	**AP (mg kg^−1^)**	**AK (mg kg^−1^)**	**C:N ratio**
**BEFORE EXPERIMENTATION IN 2007**									
Alkaline soil	8.35 ± 0.09 a	10.73 ± 1.23a	0.99 ± 0.09 a	0.43 ± 0.03 a	23.40 ± 2.02 a	78.5 ± 4.32 b	15.0 ± 2.31 a	126.0 ± 16.1 a	10.99 ± 0.14 a
Near-neutral soil	6.82 ± 0.15 b	10.43 ± 0.87 a	1.16 ± 0.11 a	0.44 ± 0.07 a	19.40 ± 1.65 b	111.0 ± 10.21 a	18.9 ± 3.08 a	97.0 ± 12.1 b	8.93 ± 0.05 c
Acidic soil	5.81 ± 0.12 c	10.06 ± 1.45 a	1.04 ± 0.09 a	0.23 ± 0.10 b	18.70 ± 1.87 b	81.2 ± 8.76 b	17.3 ± 2.98 a	133.0 ± 21.0 a	10.01 ± 0.30 b
**AFTER 7 YEARS FERTILIZATION**									
**Alkaline soil**									
M	8.31 ± 0.14 a, x	17.62 ± 1.47 a, x	1.81 ± 0.07 a, x	1.60 ± 0.13 a, x	23.81 ± 1.47 a, x	163.67 ± 16.58 a, x	126.19 ± 13.91 a, x	191.50 ± 55.50 a, x	9.71 ± 0.10 a, x
CF+M	8.12 ± 0.03 b, x	12.46 ± 0.95 b, xy	1.48 ± 0.03 b, x	1.06 ± 0.22 b, x	23.76 ± 0.95 a, x	93.25 ± 7.26 b, y	49.33 ± 8.15 b, z	112.00 ± 16.52 b, z	8.40 ± 0.26 b, y
CF	7.85 ± 0.05 c, x	10.14 ± 1.24 c, x	1.16 ± 0.03 c, x	1.03 ± 0.05 b, x	23.96 ± 1.24 a, x	76.07 ± 3.24 bc, y	33.61 ± 4.82 b, z	106.00 ± 28.00 b, x	8.77 ± 0.84 ab, x
CT	8.09 ± 0.08 b, x	10.65 ± 1.96 c, x	1.14 ± 0.05 c, x	0.73 ± 0.17 c, x	20.62 ± 1.96 a, x	67.77 ± 1.44 c, y	16.11 ± 0.19 c, z	124.50 ± 1.50 b, x	9.33 ± 0.55 ab, x
**Near-neutral soil**									
M	7.02 ± 0.11 a, y	16.86 ± 4.27 a, x	1.48 ± 0.08 a, y	1.49 ± 0.06 a, x	16.45 ± 4.27 a, y	152.13 ± 8.74 a, x	132.91 ± 5.15 a, x	314.33 ± 116.45 a, x	11.37 ± 1.05 a, x
CF+M	6.07 ± 0.14 b, y	13.91 ± 2.65 a, x	1.31 ± 0.12 b, x	1.01 ± 0.06 b, x	16.20 ± 2.65 a, y	129.06 ± 0.72 b, x	103.16 ± 4.01 b, x	204.50 ± 24.50 b, x	10.62 ± 0.23 a, x
CF	4.58 ± 0.13 c, y	9.15 ± 3.90 b, x	0.98 ± 0.01 c, y	0.99 ± 0.03 b, x	16.06 ± 3.90 a, y	128.82 ± 4.64 b, x	77.58 ± 1.82 c, x	112.67 ± 31.01 bc, x	9.36 ± 2.43 a, x
CT	5.97 ± 0.21 b, y	8.00±.20 b, y	0.81 ± 0.01 d, y	0.72 ± 0.24 cx	16.89 ± 1.20 a, xy	89.64 ± 1.50 c, x	33.91 ± 1.91 d, x	77.33 ± 10.41 c, y	9.89 ± 0.87 a, x
**Acidic soil**									
M	6.47 ± 0.25 a, z	13.93 ± 3.74 a, x	1.39 ± 0.12 a, y	1.23 ± 0.15 a, y	13.93 ± 3.74 a, z	137.95 ± 34.56*a, x*	108.09 ± 27.06 a, x	282.67 ± 35.00 a, x	10.12 ± 2.04 a, x
CF+M	5.99 ± 0.06 b, y	12.00 ± 0.13 ab, y	1.04 ± 0.11 b, y	0.94 ± 0.19 b, x	14.17 ± 0.13 a, z	119.69 ± 15.86 a, x	82.89 ± 4.45 a, y	158.50 ± 14.50 b, y	11.72 ± 1.32 a, x
CF	4.31 ± 0.13 d, y	8.63 ± 5.49 b, x	0.95 ± 0.09 b, y	0.68 ± 0.10 c, y	13.83 ± 5.49 a, z	94.93 ± 20.90 bc, y	50.60 ± 1.54 b, y	104.67 ± 23.63 b, x	8.93 ± 2.55 a, x
CT	5.88 ± 0.03 c, y	8.30 ± 0.91 b, y	0.77 ± 0.04 c, y	0.56 ± 0.06*cx*	14.25 ± 0.91 a, y	66.69 ± 3.36 c, y	22.88 ± 4.96 c, y	57.00 ± 4.00 c, z	10.85 ± 1.28 a, x
**Significance due to**									
Soil type	^***^	^*^	^***^	^**^	^***^	^***^	^***^	^*^	NS
Nutrient Source	^***^	^***^	^***^	^***^	*NS*	^***^	^***^	^***^	NS
Interaction	^***^	*NS*	^*^	*NS*	^**^	^**^	^**^	^***^	NS

*Baseline properties (mean ± SD, n = 3) before the experiment started in 2007 followed by different letters (a, b, c) denote significant differences between soil types at P = 0.05 level. Data (mean ± SD, n = 3) after 7 years' fertilization followed by different letters (a, b, c, d) or (x, y, z) denote significant (P < 0.05) differences between nutrient sources of the same soil type or between soil types with the same nutrient source, respectively*.

*, **, and *** *denote significant differences at P = 0.05, 0.01, and 0.001, respectively*.

*AK, available potassium; AN, available nitrogen; AP, available phosphorus; CF, chemical fertilizers; CF+M, chemical fertilizers plus manure; CT, control; NS, not significant; M, manure; SOC, soil organic carbon; TK, total potassium; TN, total nitrogen; TP, total phosphorus*.

To test how 7 years (2007–2014) of fertilization with contrasting nutrient sources could affect soil bacterial communities, four fertilizer treatments for each soil were examined: (1) chemical NPK fertilizers (CF), (2) manure (M), (3) CF plus M (CF+M), and (4) unfertilized control (CT). The experiment was a randomized complete block design with three replicates for each treatment. The chemical fertilizer consisted of urea, KH_2_PO_4_, and K_2_SO_4_, and the manure was a commercially composted mix of cattle manure, chicken manure, and crop straw (N 1.15, P_2_O_5_ 1.21, and K_2_O 5.34%) from the YinFu Biological Organic Fertilizer Co. Ltd., Chongqing, China. The N rates were the same for each nutrient source: 180 kg N ha^−1^ for maize and 300 kg N ha^−1^ for vegetables (50% chemical and 50% manure for the CF+M treatment). The P rate was 90 kg P_2_O_5_ ha^−1^ for both maize and vegetables, and K rates were 90 kg K_2_O ha^−1^ for maize and 150 kg K_2_O ha^−1^ for vegetables. The fertilizer treatments were applied three times during each crop phase to supply one-third of the nutrients per application. Tillage, irrigation, weeding, and plant protection practices were based on local recommended management practices.

After the Chinese cabbage harvest in December 2014, eight bulk soil cores were randomly collected (0–20 cm depth) from each replicated fertilizer treatment with a 7.5 cm diameter soil auger and thoroughly mixed to form a composite sample. After removing fine roots and visible debris, the soil was passed through a 2 mm sieve and then either air-dried for chemical analyses or stored at −80°C until DNA extraction.

### Determination of soil properties and DNA extraction

Soil pH was measured in a 1:2.5 soil to water ratio. Soil organic carbon (SOC) was determined using the K_2_Cr_2_O_7_ oxidation method (Lu, 2000). Soil total N (TN), total P (TP), and total K (TK) were determined using micro-Kjeldahl digestion, colorimetric analysis, and a dissolution-flame photometer, respectively (Lu, 2000). Soil available N (AN), P (AP), and K (AK) were determined by alkaline hydrolysis diffusion, NaHCO_3_ (0.5 mol L^−1^, pH 8.5) extraction, and NH_4_OAc (1.0 mol L^−1^) extraction, respectively (Lu, 2000). Microbial DNA was extracted from 0.5 g of freshly thawed soil using the Fast DNA SPIN Kit for Soil (Q BIOgene Inc., Carlsbad, CA, USA) according to the manufacturer's instructions. The extracted DNA was electrophoretically resolved on a 1% agarose gel to verify successful extractions which were then stored at −20°C.

### Bar-coded 454 pyrosequencing

Ten nanograms of the extracted DNA from each soil sample (one biological replicate) were used as a template for PCR amplification and subsequent Roche 454 pyrosequencing. The V1-V3 hyper-variable regions of the bacterial 16S rRNA gene were targeted using the primer set 27F (5′-AGAGTTTGATCCTGGCTCAG-3′) and 533R (5′-TTACCGCGGCTGCTGGCAC-3′). The forward primer was fused to the Roche 454 pyrosequencing adapter A (5′-CCATCTCATCCCTGCGTGTCTCCGACGACT-3′) with a 10 bp barcoded sequence for error correction and sample identification. The reverse primer was fused to adapter B (5′-CCTATCCCCTGTGTGCCTTGGCAGTCGACT-3′). The PCR was performed in 20 μL reactions (Trans gen AP221-02, TransGen Biotech, China) containing 4 μL 5 × FastPfu buffer, 2 μL of 2.5 mM dNTPs, 0.4 μL of 5 μM forward primer, 0.4 μL of 5 μM reverse primer, 0.4 μL of FastPfu polymerase and 10 ng of template DNA. Each sample was amplified in triplicate using the following procedure: 2 min of denaturation at 95°C, followed by 25 cycles of denaturation (95°C for 30 s), annealing (55°C for 30 s), extension (72°C for 1 min), and a final extension at 72°C for 10 min. The PCR products were purified using the AxyPrep DNA Gel Extraction Kit (Axygen Biosciences, Union City, CA, USA) and then quantified using the QuantiFluor™-ST (Promega, USA). A mixture of amplicons was used for pyrosequencing on a Roche 454 GS FLX+ Titanium Platform (Roche 454 Life Sciences, Branford, CT, USA) at the Majorbio BioPharm Technology Co., Ltd., Shanghai, China. The raw reads were deposited into the NCBI Sequence Read Archive (SRA) database (Accession Number SRP062251).

### Processing the pyrosequencing data

A total of 412,451 valid sequences were obtained from the 36 soil samples and were then processed with QIIME (Version 1.8.0) (Caporaso et al., 2010). Sequences with ambiguous base calls, incorrect primer sequences, average quality scores below 20 over a 50 bp sliding window, and sequences <200 bp in length with homo-polymers longer than six nucleotides were discarded. Up to two nucleotide mismatches in the primer sequences were tolerated but no mismatches in the barcode sequence were accepted. A total of 328,868 high-quality sequences were obtained with an average length of 442 bp. A total of 5,600 sequencing reads were then randomly selected from each sample using mothur (Version 1.30.1) (Schloss et al., 2011). Operational Taxonomic Units (OTUs) were clustered with 97% similarity cutoff using UPARSE Version 7.1 (http://drive5.com/uparse/, also see Edgar, 2013) and chimeric sequences were then identified and removed using UCHIME (Edgar et al., 2011). The OTUs with singleton reads were removed as they potentially originated from sequencing artifacts (Kunin et al., 2010). Representative sequences for each OTU were selected by the most abundant method using QIIME (Version 1.8.0), and the taxonomic affiliation was determined using the Ribosomal Database Project Classifier (http://rdp.cme.msu.edu/) with the Silva (SSU115) 16S rRNA database (Wang et al., 2007; Quast et al., 2012) at a confidence threshold of 70%. Rarefaction curves were generated based on the observed OTUs. Bacterial Shannon diversity index and weighted UniFrac distance based on phylogenetic information were calculated using QIIME (Version 1.8.0).

### Statistical analysis

Analysis of variance (ANOVA) was computed with SPSS 18.0 (SPSS, Inc., Chicago, IL, USA) to determine differences among fertilizer sources and soil pH at a 0.05 significance level. Regression analysis and curve fitting were performed with SigmaPlot 12.5 (Systat Software, Inc., San Jose, CA, USA) to assess relationships between the relative abundance of different major bacterial phyla and soil pH. A heatmap based on the relative abundances of bacterial phyla was generated using the Bray-Curtis Distance Calculation and Hierarchical Cluster Analysis with the vegdist and hclust functions of the R package “vegan.” Principal Coordinate Analysis (PCoA) based on the weighted Unifrac distance were used to visualize the bacterial community structures in relation to each fertilization treatment for each soil. Permutational Multivariate Analysis of Variances (PERMANOVA) based on weighted Unifrac distance were performed to analyze the effect of fertilization or soil type on bacterial communities, in which the “strata” option in the R package “vegan” was used to constrain the fertilization or soil type factor within each soil or nutrient source (Anderson, 2001). Distance-based Redundancy Analysis (dbRDA) and marginal tests based on weighted Unifrac distances were performed to estimate variability in the bacterial community structure explained by soil chemical properties. The Heatmap, PCoA, and dbRDA were computed using the Vegan package in R (Oksanen et al., 2013; R Development Core Team, 2013).

## Results

### Soil chemical properties

Soil pH, SOC, TN, TP, AN, AP, and AK were all significantly (*P* < 0.05) affected by nutrient source, but TK and the C:N ratio were not (Table 1). For each soil type, AP and AK were significantly increased after 7 years of fertilization, while the increase in AN was of a smaller magnitude compared with the initial AN concentration in each soil. Fertilization with M led to the greatest AP and AK values, which were 29–275% and 54 to 179% greater than those in the CF and CF+M treated soils, respectively. The AP accumulated most in the manure-amended alkaline soil compared with the other nutrient sources, while AK accumulated most in the manure-amended near-neutral and acidic soil compared with the CF treatments.

Fertilizing with M led to the highest pH values, followed by CF+M, CT and then CF, regardless of the initial soil pH (Table 1). In the alkaline soil, fertilization with M increased soil pH by 0.5 and 0.2 units compared with CF and CT treatments, while in both the acidic and near-neutral soil, fertilization with M increased soil pH by 2.2 and 2.4 units compared with CF treatments and 0.6 and 1.1 units compared with CT treatments, respectively. Overall, soil pH in the acidic and near-neutral soil was similar under CF+M, CF, and CT (Table 1), but soil pH was greater in the M-amended near-neutral soil than in the M-amended acidic soil.

### Bacterial community structure

**Figure 1:**
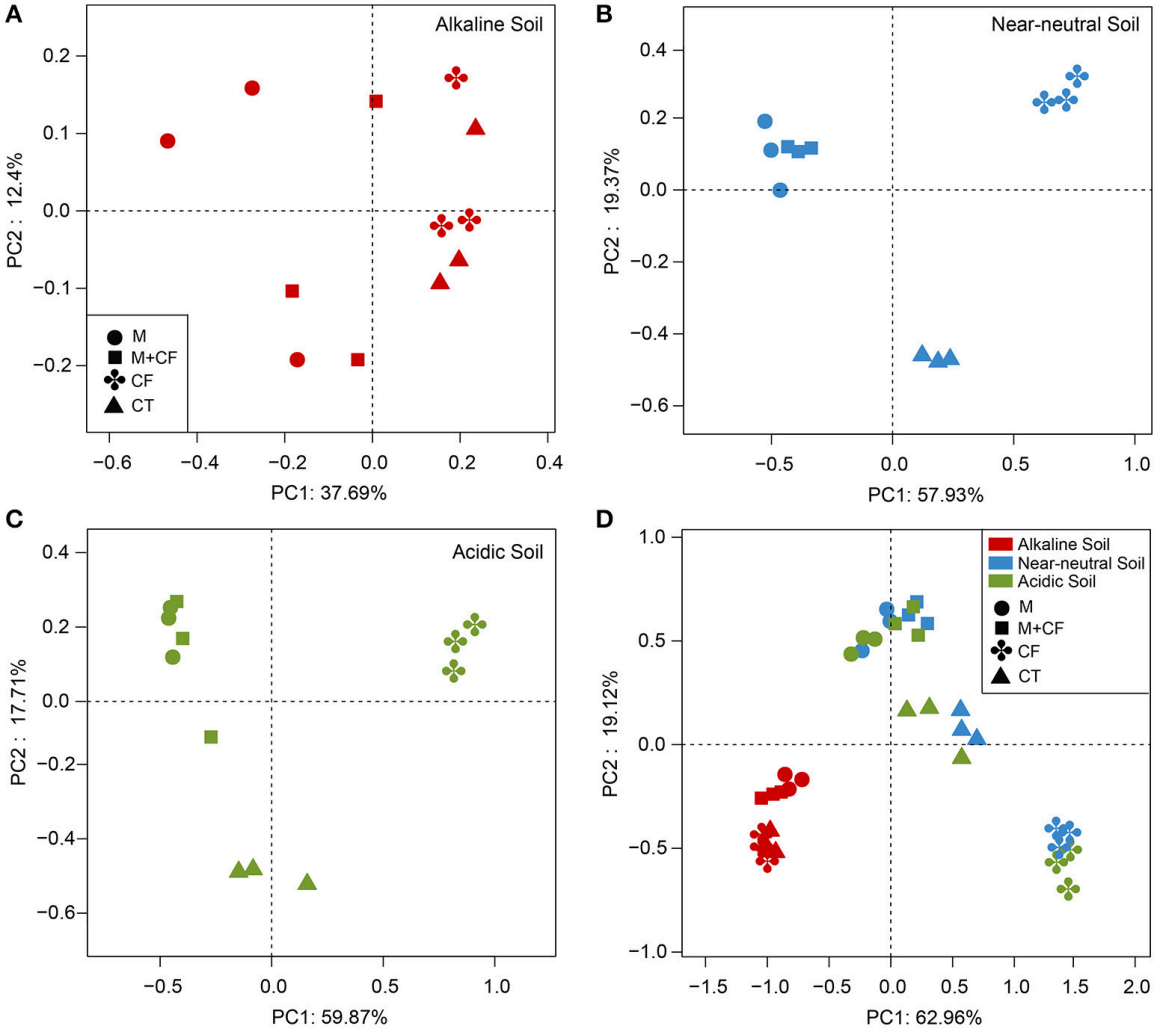
Principal coordinate analysis of bacterial community structure in the alkaline soil **(A)**, near-neutral soil **(B)**, acidic soil **(C)**, and the three soils combined **(D)**. Principal Coordinate Analysis (PCoA) based on the weighted Unifrac distance. CF, chemical fertilizers; CF+M, chemical fertilizers plus manure; CT, control; M, manure.

Although the nutrient source significantly affected bacterial community structure in the alkaline soil (Table 2), the bacterial community structures were not distinctly grouped by a nutrient source in the PCoA plot (Figure 1A). However, in the acidic and near-neutral soils, the bacterial community clustered into three groups according to nutrient sources that had been applied for 7 years (M and CF+M, CT, and CF), which explained 57.93% and 59.87% of the variance along the first axis on the PCoA plots (Figures 1B,C), respectively. These effects were also supported by the PERMANOVA (Table 2), which demonstrated that there were significant differences among nutrient sources in acidic and near-neutral soils. Across the three soils tested, the bacterial communities were clustered into four groups on the PCoA plot: all nutrient sources in the alkaline soil, the M and CF+M, the CT and the CF in the acidic and near-neutral soil (Figure 1D). The bacterial community structure of CF in acidic and near-neutral soil were clustered very closely while distant from other nutrient sources (Figure 1D), suggesting a marked effect of mineral fertilizer on the bacterial community structure in the acidic and near-neutral soils. These results were also linked with the nutrient source PERMANOVA test (Table 2). The dbRDA model examined the relationship between soil chemical properties and the bacterial community structure (Table 3). In the acidic and near-neutral soil, soil pH was the strongest predictor of bacterial community structure, explaining 11.64% (*P* = 0.02) and 18.28% (*P* = 0.001) of the variance, respectively (Table 3). In contrast, bacterial community structure was not significantly affected by the chemical properties of the alkaline soil (Table 3).

**Table 2:** Permutational multivariate analysis of variance (PERMANOVA) exploring the differences in bacterial community structure among nutrient source or soil types.

**Tested factors**	***F***				***P-*****value**	
**Nutrient source**	3.92				0.001	
**Soil type**	9.22				0.001	
	Alkaline soil		Near-neutral soil		Acidic soil	
	***F***	***P-*****value**	***F***	***P-*****value**	***F***	***P-*****value**
Nutrient source	1.93	0.001	6.92	0.001	6.46	0.001

*Effects of nutrient source or soil type on bacterial community structure were determined by multivariate permutational analysis of variance (PERMANOVA). Values represent the pseudo-F ratio (F) and the level of significance (P)*.

**Table 3:** Relationships between soil chemical properties and bacterial community structure.

	**pH VC (*P*)**	**AN VC (*P*)**	**AP VC (*P*)**	**AK VC (*P*)**	**TN VC (*P*)**	**TP VC (*P*)**	**TK VC (*P*)**	**SOC VC (*P*)**	**C:N VC (*P*)**
Acidic soil	**18.28%**	4.01%	4.97%	3.52%	5.35%	4.65%	3.03%	5.65%	5.40%
	**(0.008)**	(0.47)	(0.35)	(0.53)	(0.32)	(0.37)	(0.64)	(0.28)	(0.30)
Alkaline soil	8.58%	7.46%	8.50%	8.49%	7.19%	6.16%	5.73%	7.16%	7.10%
	(0.15)	(0.24)	(0.15)	(0.15)	(0.26)	(0.39)	(0.46)	(0.27)	(0.27)
Near-neutral soil	**11.64%**	4.16%	4.23%	4.19%	4.18%	4.75%	3.64%	4.22%	4.25%
	**(0.02)**	(0.24)	(0.24)	(0.24)	(0.26)	(0.22)	(0.31)	(0.25)	(0.25)
Total soils	**9.34%**	1.74%	2.22%	1.44%	1.40%	1.25%	2.17%	1.00%	1.08%
	**(0.001)**	(0.19)	(0.12)	(0.29)	(0.27)	(0.37)	(0.12)	(0.47)	(0.44)

*The variance component (VC) for each chemical property to explain soil bacterial community structure was performed by distance-based redundancy analysis (dbRDA). Significant P-values were performed by marginal tests and are shown in parentheses. P < 0.05 are indicated in bold*.

### Bacterial richness and diversity

**Figure 2:**
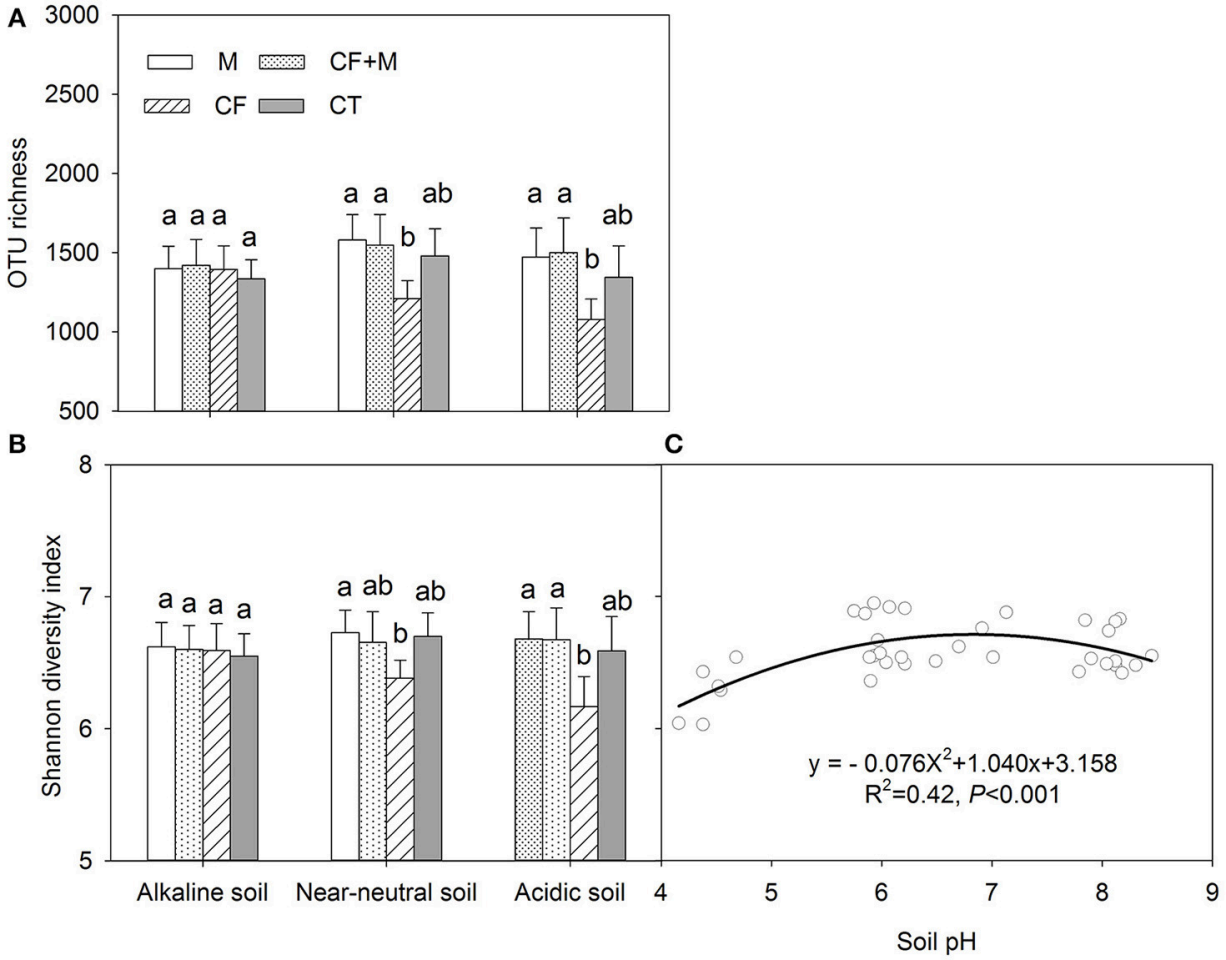
Variation of the number of operational taxonomic units (OTU richness) **(A)** and Shannon diversity index **(B)** of soil bacterial communities and the non-linear relationship between soil pH and Shannon diversity index **(C)** Data (mean ± *SD, n* = 3) followed by different letters (a, b) denote significant differences between nutrient sources in the same soil type at *P* = 0.05. CF, chemical fertilizers; CF+M, chemical fertilizers plus manure; CT, control; M, manure. The solid line is the regression fitting curve between soil pH and bacterial Shannon index.

The rarefaction curve indicated variation in OTU density within the soil samples, while the sequence coverage sufficiently captured the bacterial community diversity (Figure S1). The nutrient source did not significantly affect bacterial OTU richness and the Shannon diversity index in the alkaline soil (Figures 2A,B). In the acidic and near-neutral soil, bacterial OTU richness and Shannon diversity index were slightly increased with CF+M and M, while slightly decreased by CF, compared with CT (Figures 2A,B). Interestingly, both the soil bacterial OTU richness and Shannon diversity index were significantly (*P* < 0.05) lower with CF than M and CF+M in the near-neutral and acidic soils, except for the Shannon diversity index in the near-neutral soil (Figures 2A,B). The bacterial Shannon diversity index increased from acidic to near-neutral pH and slightly decreased from near-neutral to alkaline pH (Figure 2C).

### Bacterial community composition

Overall, sequences were assigned to 30 bacterial phyla (Figure S2). The most dominant phyla were Proteobacteria (mean relative abundance: 24.6%), Actinobacteria (19.7%), Chloroflexi (15.3%) and Acidobacteria (12.6%); the medium dominant phyla were Bacterioidetes (5.3%), Planctomycetes (4.8%), Gemmatimonadetes (4.5%), Firmicutes (3.4%), Cyanobacteria (2.1%), Nitrospirae (1.8%) and candidate division TM7 (1.0%). Altogether, these phyla accounted for more than 95% of the total bacterial relative abundance (Figure S2). The least abundant phyla were Verrucomicrobia (0.7%), Armatimonadetes (0.6%), candidate division WS3 (0.4%) and Fibrobacteres (0.3%).

The bacterial community composition clustered into three groups based on the Bray-Curtis distance calculation and the hierarchical cluster analysis on the Heatmap (Figure S2). In general, the bacterial community composition receiving CF or no nutrient inputs (CT) was closely clustered and showed little similarity with other treatments in the acidic and near-neutral soil. A similar bacterial community composition was observed with M and CF+M in the acidic and near-neutral soils, while all nutrient sources had similar bacterial community composition in the alkaline soil.

### Relationships between soil pH and dominant bacterial phyla

**Figure 3:**
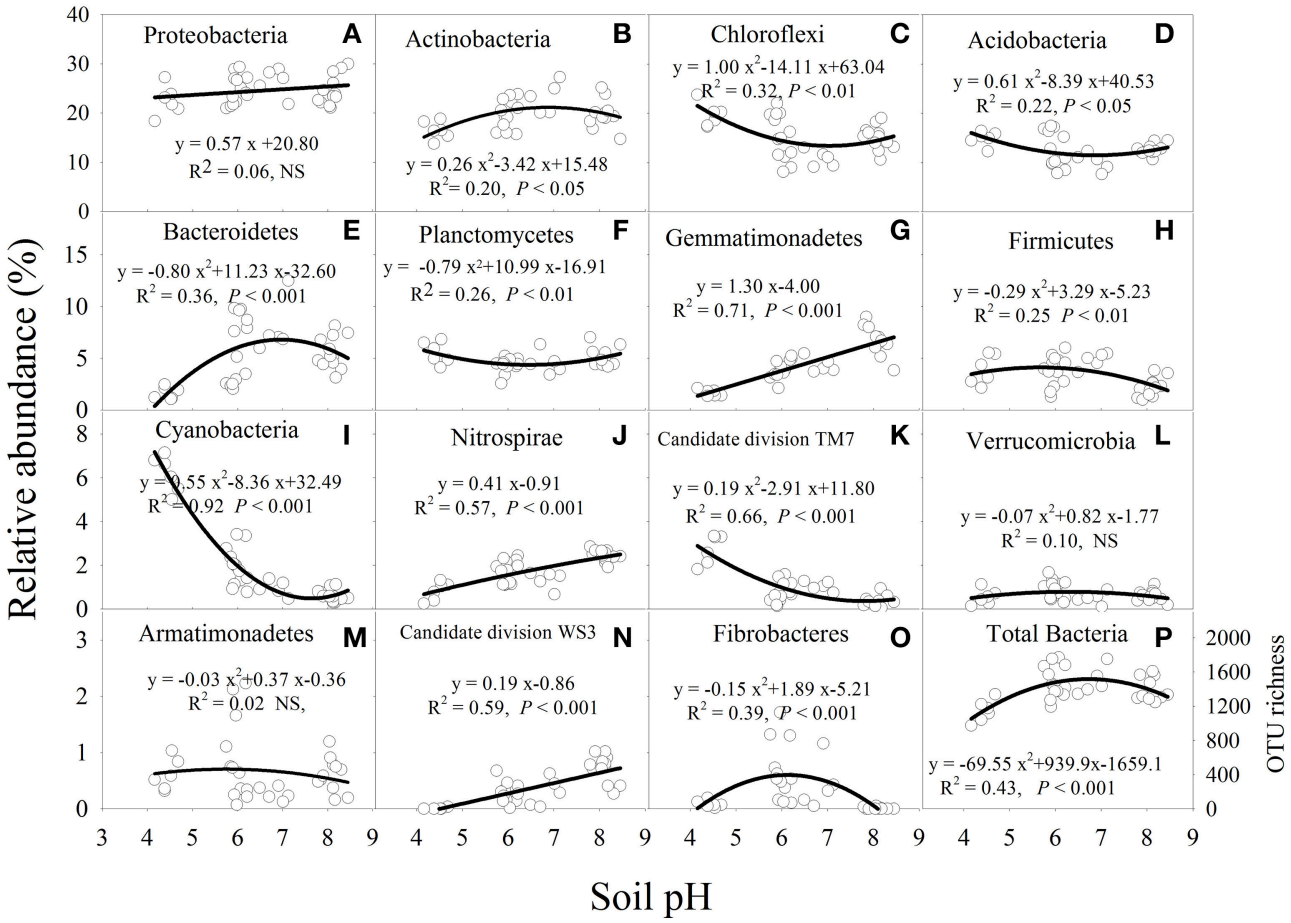
Relationships between soil pH and the relative abundance of each bacterial phylum from a total of 15 major bacterial phyla **(A–O)** and total bacterial OTU richness **(P)** after 7 years of chemical fertilizer and/or manure applications. NS, not significant; OTUs, operational taxonomic units. Solid lines are regression fitting curves between soil pH and relative abundance of each bacterial phylum.

Soil pH had a significant (*P* < 0.05) influence on the relative abundance of the major bacterial phyla, except for Armatimonadetes, Proteobacteria, and Verrucomicrobia (Figure 3). The relative abundance of Actinobacteria, Bacterioidetes, Fibrobacteres, and Firmicutes was higher at near-neutral pH and lower at acidic and alkaline pH (Figures 3B,E,H,O). In contrast, the relative abundance of Acidobacteria, Chloroflexi or Planctomycetes decreased from acidic to near-neutral pH and then increased from near-neutral to alkaline pH (Figures 3C,D,F). In addition, the relative abundance of Gemmatimonadetes, Nitrospirae or candidate division WS3 was linearly increased with soil pH (Figures 3G,J,N), while Cyanobacteria or candidate division TM7 decreased in a quadratic manner as soil pH increased (Figures 3I,K). The bacterial OTU richness increased from acidic to near-neutral pH and slightly decreased from near-neutral to alkaline pH (Figure 3P).

## Discussion

### Application of chemical or organic fertilizer altered soil pH

Application of chemical, organic, or chemical plus organic fertilizers altered soil nutrient concentrations and pH (Table 1), and these changes were associated with distinct shifts in the bacterial communities. Soil pH was decreased by 1.6 and 1.4 units after applying CF for 7 years to the acidic and near-neutral soils, compared with the unfertilized CT, respectively (Table 1). The reduced soil pH was probably due to an excessive urea application to soil (Guo et al., 2010; Schroder et al., 2011; Tian and Niu, 2015). When urea is applied to soil, it is rapidly hydrolized by urease to release ammonium (${\text{NH}}_{4}^{+}$). The ${\text{NH}}_{4}^{+}$ is then quickly oxidized by microorganisms in three stepwise reactions to hydroxylamine, nitrite and then nitrate, a process that releases two H^+^ ions (Barak et al., 1997). A smaller decrease in soil pH (−0.2 units) was observed under CF in the alkaline soil, compared with the CT (Table 1). This likely resulted from the higher carbonate content in the alkaline soil, which has a strong buffering capacity against acidification. In addition, soil pH in all three soils was significantly (*P* < 0.05) or noticeably increased with manure (M and CF+M), but significantly decreased with CF applications compared with the CT (Table 1). Studies have shown that soil acidification may be alleviated or soil pH increased by applying organic residues because they release hydroxyl ions (OH^−^) during decomposition (Bolan et al., 1991; Naramabuye and Haynes, 2006; Rashid et al., 2013; Cai et al., 2015; Sun et al., 2015). The applications of manure to acidic soil may reduce exchangeable and soluble Al concentrations (Naramabuye and Haynes, 2006) and increase soil pH to near neutral (Whalen et al., 2000).

### Soil pH drives soil bacterial community structure

There is limited understanding of how soil nutrient availability and pH may interact to shape bacterial community structure (Geisseler and Scow, 2014). The application of contrasting fertilizer sources to acidic, near-neutral or alkaline Eutric Regosol soil allowed us to assess the direct effect of nutrient inputs (in alkaline soil), and the interactive effect of pH and nutrient availability (in acidic and near-neutral soil) on soil bacterial communities.

In our study, the alkaline soil was apparently buffered against changes in soil pH, which was maintained between 7.79 and 8.45. Such a narrow soil pH range may provide a condition to investigate nutrient input effects on bacterial diversity without considering the effect of altered pH. In the alkaline soil, fertilization with M and CF+M significantly increased soil AN, P, K, and SOC, compared to CT (Table 1), but no significant differences in bacterial OTU richness and Shannon diversity index were observed (Figures 2A,B). Moreover, PCoA ordination analysis indicated a smaller distinction between bacterial community structure with the manure-based fertilizers and non-manured treatments in the alkaline soil (Figure 1A) than in the acidic and near-neural soils (Figures 1B,C). No clear relationships were observed between soil chemical properties and bacterial community structure in the alkaline soil (Table 3). These results are inconsistent with previous studies that found amending soil with a diverse organic substrate, such as, manure increased bacterial diversity and led to a more distinct bacterial community structure than the chemical fertilizer (Hartmann et al., 2015; Sun et al., 2015; Francioli et al., 2016). Such inconsistencies may arise from the experimental conditions, especially differences in soil pH. For instance, the alkaline soil pH values were consistently above 7.79 (Table 1), whereas previous studies were typically conducted with soil pH ranges between 5.40 and 7.05 (Hartmann et al., 2015; Sun et al., 2015; Francioli et al., 2016).

In the acidic and near-neutral soils, bacterial OTU richness, Shannon diversity index, and soil pH were significantly decreased with CF, compared to M and CF+M applications (Table 1, Figures 2A,B). This was consistent with the finding that declining soil pH, which was induced by chemical fertilization, decreased soil bacterial richness and diversity and that applying cow or pig manure could restore soil pH and bacterial diversity to levels similar to non-chemical-amended soil (Sun et al., 2015).

It is challenging to distinguish the direct effect of nutrient input and indirect effect of altered pH on bacterial community structure when soil nutrient availability and pH are simultaneously altered by fertilization. A number of studies in different soil types have reported that bacterial community structure is mainly determined by soil pH (Fierer and Jackson, 2006; Rousk et al., 2009, 2010; Siciliano et al., 2014; Sun et al., 2015; Zhalnina et al., 2015). A similar result was found in the present study, whereby across the soil pH gradient, soil pH was the strongest predictor of bacterial community structure and had a greater effect on bacterial community structure than nutrient availability (Table 3). When the same nutrient source was applied for 7 years to the acidic, near-neutral and alkaline soils, an apparently distinct bacterial community composition (Figure S2) and community structure (Figure 1D) were observed between the alkaline soil, and acidic and near-neutral soils, which corresponded with distinct soil pH values (Table 1). It seems that the other edaphic properties instead of soil pH have relatively small impacts on soil bacterial community composition and structure, which is consistent with previous studies that reported soil pH was a continental scale control on soil bacterial community structure (Fierer and Jackson, 2006; Lauber et al., 2009).

Moreover, in acidic or near-neutral soils, soil bacterial community composition (Figure S2) and structure (Figures 1B,C) were apparently distinct between manure-based fertilization (M and M+CF) and CF, which corresponded with mutually exclusive soil pH ranges (5.93–7.13 in the M and M+CF vs. 4.16–4.68 in the CF; Table 1). However, soil pH values receiving M and M+CF (5.93–7.13), and the CT (5.75–6.18) (Table 1) in acidic and near-neutral soils somewhat overlapped, but the bacterial community structures were significantly distinct (Figures 1B,C). This indicates that soil pH was likely the main driver, but other factors such as, nutrient availability or substrate quality also shapes bacterial community composition and structure. Soil C and N are predominant limiting factors for bacterial growth. Manure application significantly increased soil organic C and N (Table 1) compared with the CT and CF treatments; this may have promoted soil microbial activity (Ninh et al., 2015) and hence altered soil bacterial community structure (Cookson et al., 2005; Hartmann et al., 2015).

### Responses of bacterial community composition to soil pH

Although Rousk et al. (2010) reported that both bacterial OTU richness and 16S rRNA gene copy numbers increased linearly between soil pH 4.0 and 8.3, the present study shows both total bacterial OTU richness and Shannon diversity index increased from acidic to near-neutral pH and decreased from near-neutral to alkaline pH (Figures 2C, 3P). Similar negative quadratic responses of bacterial phylotype richness and diversity to increasing soil pH (~3.5 to 8.8) have been reported (Fierer and Jackson, 2006; Lauber et al., 2009). The sensitivity of poorly adapted bacterial taxa to pH changes was considered to be the main reason for the decline of bacterial diversity in acidic and alkaline soils (Lauber et al., 2009; Zhalnina et al., 2015). In the present study, there were no apparent differences in soil bacterial OTU richness and Shannon diversity index when soil pH ranged from 6.0 to 8.5 (Figures 2C, 3P). This suggests that the optimal pH range of bacterial diversity extends beyond 8.0 in some soils. Manure application likely prevented soil acidification and thus buffered against potential effects on soil bacterial richness and diversity. In contrast, chemical fertilizers acidified the near-neutral and acidic soil to pH < 6.0, thereby reducing bacterial richness and diversity (Figures 2A,B).

The narrow pH range for optimal bacterial growth may be the primary factor influencing bacterial community composition and structure (Lauber et al., 2009; Rousk et al., 2010). The majority of bacteria require an intracellular pH between 6.0 and 8.0 (Fierer and Jackson, 2006; Zhang et al., 2015), which is suitable for most proteins to function (Krulwich et al., 2011). Our results showed that most soil bacteria groups were more abundant in near-neutral soil than in acidic and alkaline soils (Figure 3). The relative abundance of Actinobacteria, Bacteroidetes, Fibrobacteres, and Firmicutes increased from acidic to near-neutral pH and decreased from near-neutral to alkaline pH (Figure 3). A greater relative abundance of Actinobacteria in near-neutral pH was observed in a broad range of ecosystems (Lauber et al., 2009). Wu et al. (2017) also found Bacteroidetes were most abundant in near-neutral pH agricultural soils. On the contrary, Chu et al. (2010) reported that Actinobacteria and Bacteroidetes had a positive linear response with increasing soil pH (~4.0 to 7.8). It is possible that such observed differences may be attributed to edaphic properties or environmental conditions (e.g., temperature). Another reason may be that the soil samples were from subtropical agroecosystems in the present study, but were from Arctic non-agricultural soils in the study by Chu et al. (2010). Cyanobacteria groups have been reported as important N-fixing microorganisms (Vitousek et al., 2002). In our study, the bacterial phyla of Cyanobacteria were most abundant in the acidic soil (Figure 3). The higher abundance of Cyanobacteria was mainly attributed to the lower availability of soil N (Ramirez et al., 2010), and a lower TN was observed in acidic than in alkaline soil in this study (Table 1). The phyla of Gemmatimonadetes and Nitrospirae were vulnerable to soil acidity and were most abundant in the alkaline soil (Figure 3). Similar significant and positive relationships between pH and the relative abundance of Nitrospirae were also reported by previous studies (Liu et al., 2014; Zhalnina et al., 2015; Wu et al., 2017). Although Zhalnina et al. (2015) reported that the relative abundance of Gemmatimonadetes was positively correlated with soil pH, which was consistent with this study, some studies found that there was no significant relationship between soil pH and Gemmatimonadetes abundance (Liu et al., 2014; Wu et al., 2017). These inconsistent findings are probably because some bacterial groups may show a site-specific response to soil pH. For instance, bacterial taxa that are prevalent in acidic or alkaline soils may have evolved to cope with a reduced extracellular enzyme activity and slower microbial cell metabolism (Waldrop and Zak, 2006).

## Conclusion

Applications of chemical fertilizers, composted manure or their combination generally increased soil nutrient availability, but also significantly altered soil pH, especially in the acidic and near-neutral soils. The nutrient source had no detectable effects on bacterial richness, composition, and diversity in the alkaline soil, but chemical fertilizers significantly reduced bacterial richness and diversity in the acidic and near-neutral soil, compared with composted manure and the combination of chemical fertilizer and composted manure. Most bacterial phyla were more abundant in near-neutral or alkaline soil than in the acidic soil, which suggested that soil acidification induced by chemical fertilizers decreased total bacterial richness and diversity. Thus, this study showed that fertilizer applications indirectly affected soil bacterial diversity by changing soil pH more than through the direct input of nutrients to soil. To reduce soil acidification and its negative effects on most soil bacteria, organic fertilizers alone or in combination with chemical fertilizers are recommended as nutrient sources.

## Author contributions

XjS designed the study and contributed the management and maintenance of long-term field experiment. YtZ collected soil samples, contributed to the physicochemical soil analysis and analyzed the sequencing data. YtZ, HS, XhH, BT, NL, XyH, MT and XjS wrote the manuscript.

### Conflict of interest statement

The authors declare that the research was conducted in the absence of any commercial or financial relationships that could be construed as a potential conflict of interest.
