# Supplementary material for: Fertilization Shapes Bacterial Community Structure by Alteration of Soil pH
Source: Front Microbiol. 2017 Jul 18;8:1325. doi: 10.3389/fmicb.2017.01325 (PMC5513969; doi:10.3389/fmicb.2017.01325)
Supplement: Supplementary file 1 [file Presentation1.PPT]

## Slide 1
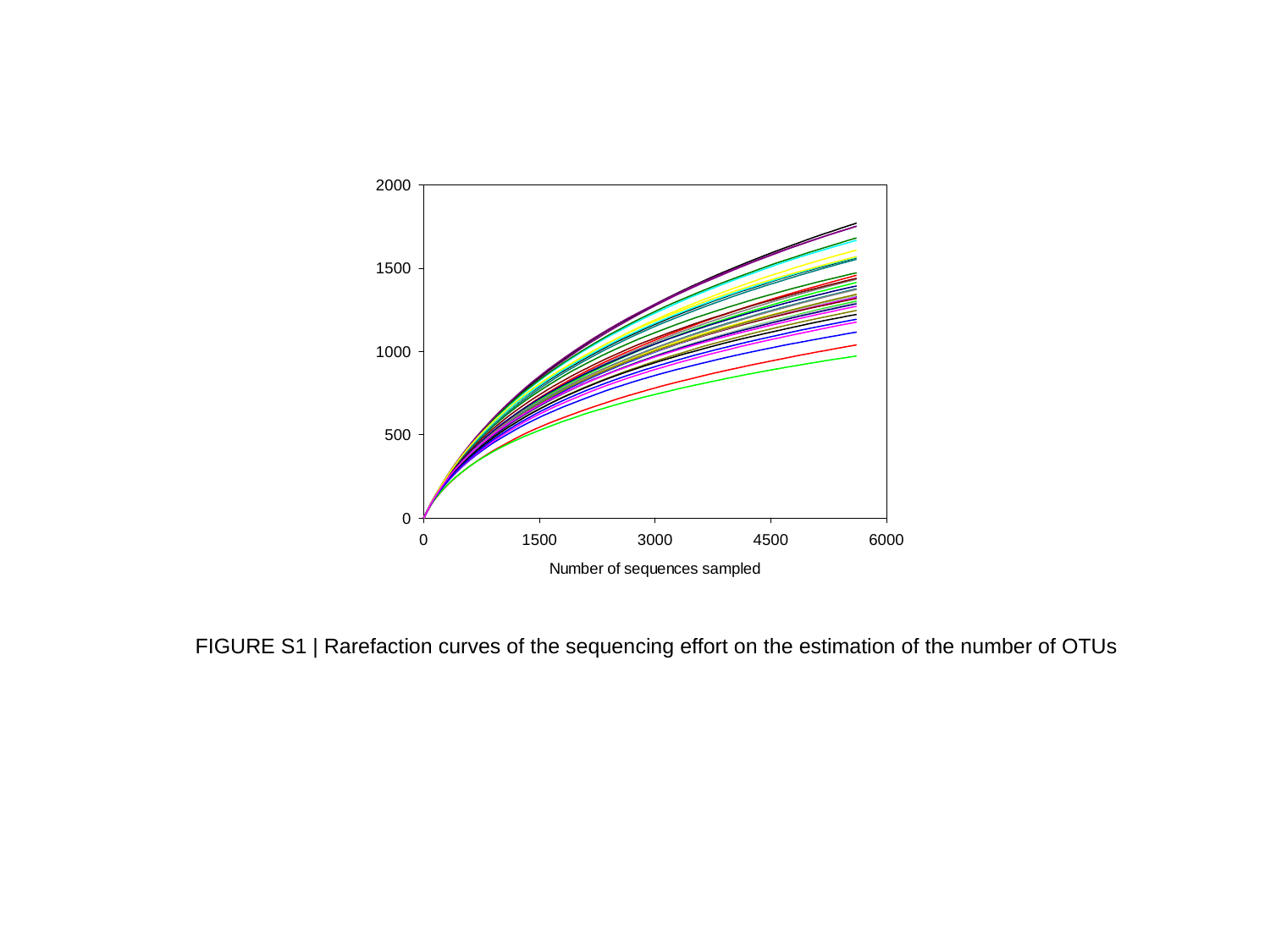

FIGURE S1 | Rarefaction curves of the sequencing effort on the estimation of the number of OTUs
