# Supplementary material for: Fertilization Shapes Bacterial Community Structure by Alteration of Soil pH
Source: Front Microbiol. 2017 Jul 18;8:1325. doi: 10.3389/fmicb.2017.01325 (PMC5513969; doi:10.3389/fmicb.2017.01325)
Supplement: Supplementary file 2 [file Presentation2.PPT]

## Slide 1
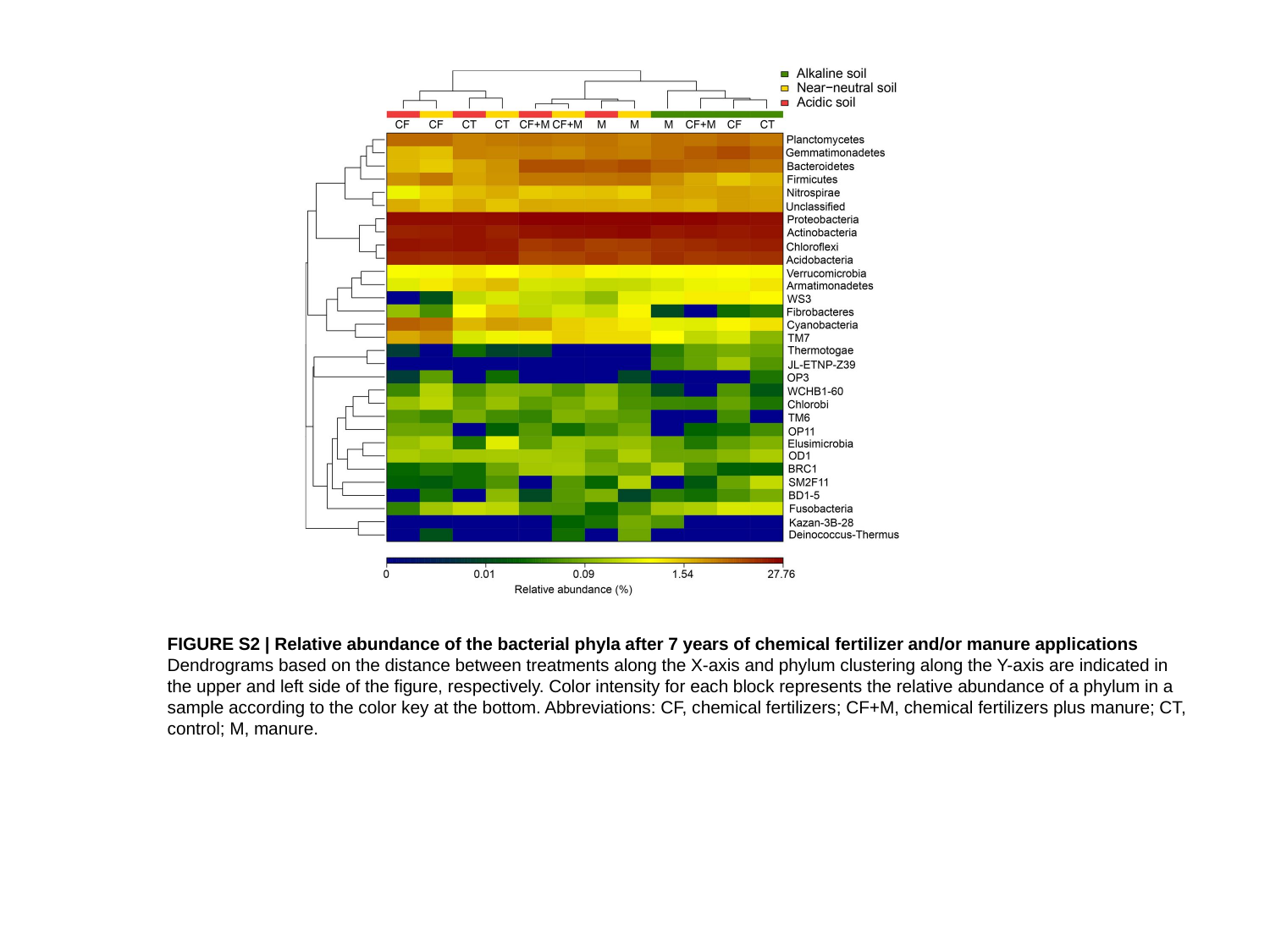

FIGURE S2 | Relative abundance of the bacterial phyla after 7 years of chemical fertilizer and/or manure applications
Dendrograms based on the distance between treatments along the X-axis and phylum clustering along the Y-axis are indicated in the upper and left side of the figure, respectively. Color intensity for each block represents the relative abundance of a phylum in a sample according to the color key at the bottom. Abbreviations: CF, chemical fertilizers; CF+M, chemical fertilizers plus manure; CT, control; M, manure.
